# Epigallocatechin-3-gallate suppresses differentiation of adipocytes via regulating the phosphorylation of FOXO1 mediated by PI3K-AKT signaling in 3T3-L1 cells

**DOI:** 10.18632/oncotarget.23590

**Published:** 2017-12-21

**Authors:** Yi Lu, Junye Chen, Tao Xian, Yumeng Zhou, Wanwan Yuan, Mengxi Wang, Yuyang Gan, Kun Wang, Shaofeng Xiong, Cong Ma, Xueying Yu, Qiren Huang

**Affiliations:** ^1^ Key Provincial Laboratory of Basic Pharmacology, Nanchang University, Nanchang 330006, Jiangxi Province, P.R. China; ^2^ Department of Pharmacology, School of Pharmacy, Nanchang University, Nanchang 330006, Jiangxi Province, P.R. China; ^3^ Jiangxi Medical School, Nanchang University, Nanchang 330006, Jiangxi Province, P.R. China; ^4^ Nanchang Joint Programme, Queen Mary University of London, London E1 4NS, UK

**Keywords:** epigallocatechin-3-gallate, adipogenesis, adipocyte, forkhead box-O1, peroxisome proliferator activated receptor γ

## Abstract

Epigallocatechin-3-gallate (EGCG) is a pivotal effective component of green tea. It is known that EGCG has antioxidant activity, anti-angiogenesis, anti-tumor, cardiovascular protection and blood lipid regulation functions. Forkhead box-O1 (FOXO1) is one of the downstream signals of protein kinase B (AKT) and takes part in adipogenesis. The purpose of this study is to investigate the effects of EGCG on adipose differentiation and the likely mechanisms. 3T3-L1 cells were induced by DMI for 2, 4, 6 and 8 days, respectively. During induction, the cells were treated with EGCG (5 μM, 10 μM, 50 μM and 100 μM) or DMSO for the first 2 days. In addition, another batch of 3T3-L1cells were treated with SC-3036 (PI3K activator, 10 µM), or LY294002 (PI3K inhibitor, 10 µM) alone or combined with EGCG (100 μM) for the indicated times. Medium glucose concentration, lipid accumulation, the levels of TNF-α, resistin, adiponectin and leptin and the expression of FOXO1, phosphorylated-FOXO1 (P-FOXO1), PPARγ, fatty acid synthase (FAS) were detected, respectively. The present study demonstrated that EGCG inhibited glucose uptake, lipid accumulation and adipokine secretion in a concentration-dependent manner during adipogenesis, which suggests that EGCG inhibits adipocyte’s differentiation, maturation and functions. Moreover, EGCG also down-regulated the expression levels of PPARγ and P-FOXO1. Conversely, the PI3K activator reversed these changes caused by EGCG, suggesting that the inhibitory effects of EGCG may be mediated by PI3K-AKT-FOXO1 pathway to negatively regulate the expression of PPARγ. The findings will provide a solid foundation for EGCG to prevent and cure the obesity-associated diseases.

## INTRODUCTION

Obesity is caused by an increase in fat due to the imbalance of energy intake and consumption [1]. In the modern world, people suffering from obesity and overweight are on a rise because of high-fat and high-calorie diets and sedentary lifestyle [2, 3]. Incidence of the diseases triggered by obesity is growing and the degree of damage is also becoming increasingly severe. The diseases associated with obesity include insulin resistance, fatty liver disease, type 2 diabetes, atherosclerosis, hypertension, stroke, arthritis, and numerous cancers [4, 5]. Adipose tissue used to be considered as an energy-saving organ, but currently is recognized as an endocrine organ [6]. Adipose tissue can excrete a variety of bioactive polypeptides known as adipokines, including leptin, adiponectin, resistin, TNF-α, IL-6 and so on [7–10]. Adipokines regulate adipocyte differentiation, metabolism and local inflammatory reaction via autocrine and paracrine pathways, and play important roles in insulin resistance and chronic inflammation process [11, 12].

Epigallocatechin-3-gallate (EGCG) is a primary potent component of green tea, which can be extracted from green tea leaves [13]. Its roles in antioxidant activity, anti-angiogenesis, anti-tumor, cardiovascular protection and blood lipid regulation are research hotspots [13–17]. In addition, several studies found that EGCG has an inhibitory effect on adipogenesis. In 2002, Prusty D. *et al*. have demonstrated that EGCG promotes adipocyte apoptosis and inhibits adipogenesis [18]. Watanabe J. *et al.* have also proved that EGCG suppresses adipocyte differentiation via inhibiting the accumulation of lipid in 3T3-L1 mature adipocyte and the activation of rate-limiting enzyme and acetyl-CoA carboxylase, of fatty acid biosynthesis [19]. Wolfram S *et al.* have showed that 100 μM EGCG markedly inhibits the proliferation of 3T3-L1 preadipocyte [20]. Liu *et al.* considered that EGCG inhibits 3T3-L1 adipocyte differentiation and resistin gene expression through extracellular signal-related kinases (ERKs) in mitogen-activated protein kinase (MAPK) pathway and phosphatidylinositol 3-kinase (PI3K) pathway [21]. Meanwhile, EGCG can inhibit the phosphorylation of ERK1/2, which down-regulates the expression of PPARγ.

Forkhead box-O1 (FOXO1) is a member of the FOXO family which comprises FOXO1, FOXO3, FOXO4 and FOXO6 [22]. FOXO1, a transcription factor, is one of the downstream signals of AKT and takes part in cell cycle control, apoptosis, metabolism, etc. [23–26]. FOXO1 is normally expressed at insulin responsive tissues, such as liver, adipose tissue, pancreas and so on [24–26]. Consequently, it is also drawn extensive attention in cancer researches [26–28]. Likewise, a number of researchers have demonstrated that it is also involved in the regulation of adipocyte differentiation. During adipogenesis, FOXO1 binds to the promoter of PPARγ to inhibit the transcriptional activity of PPARγ [29]. Overall, these data suggest that FOXO1 plays an essential role in adipocyte differentiation. Normally, FOXO1 stays in nucleus and maintains its transcriptional activity. Once phosphorylated, it enters cytoplasm from nucleus and loses its transcriptional activity [22, 30]. FOXO1 has three highly conserved phosphorylation sites in human, i.e., Thr24, Ser256 and Ser319 which are phosphorylated by AKT [31]. Our previous research has also confirmed that EGCG inhibits the differentiation of adipocytes via inhibition of AKT phosphorylation and decreases the expression levels of PPARγ [32]. This study is further to investigate the effects and mechanism of EGCG on adipogenesis and adipocyte functions.

## RESULTS

### EGCG suppresses the glucose uptake during adipogenesis

**Figure 1:**
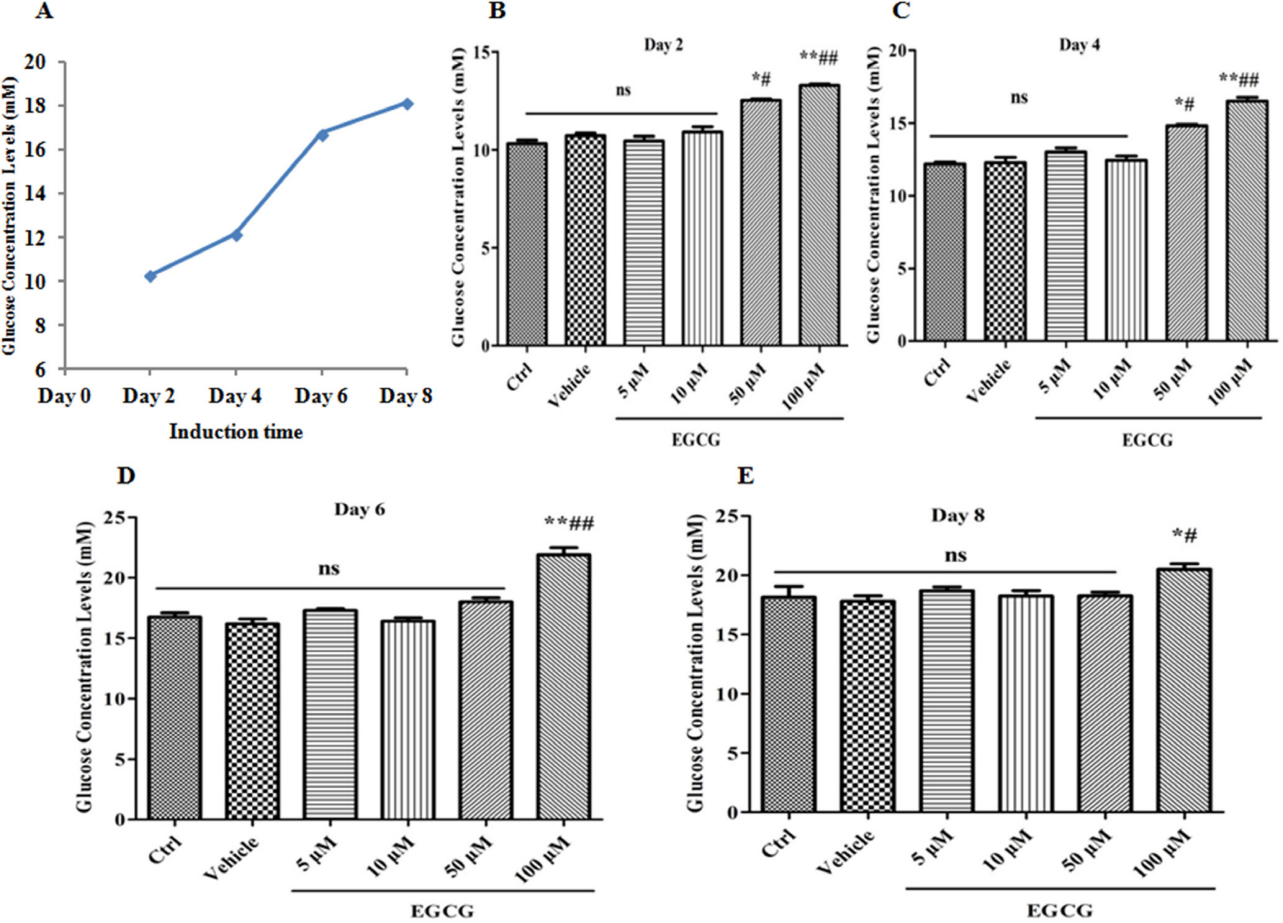
EGCG suppresses the glucose uptake during adipogenesis 90% confluent 3T3-L1 preadipocytes were induced by DMI for 2, 4, 6 and 8 days, respectively. During induction, the cells were treated with EGCG (5 μM, 10 μM, 50 μM and 100 μM) or DMSO for the first 2 days (**A–E**). Medium glucose concentrations (mM) were detected with a glucose concentration assay kit. Error bars show the S.E.M. of 3 independent experiments. ^*^P < 0.05, ^**^P < 0.01, vs. Ctrl or Vehicle group; ^#^*P* < 0.05, ^##^*P* < 0.01, vs. EGCG (5 μM). Ctrl: Control, Vehicle: DMSO, ns: no significance.

As shown in our previous data, undergone induction for 8 days, 3T3-L1 preadipocytes nearly differentiated to mature adipocytes [32]. Our present results showed that with elongation of induction time, the medium glucose concentrations gradually increased, demonstrating that preadipocytes consume more glucose than mature adipocytes (Figure 1A). On day 2 and day 4 of induction, treatment with EGCG (5 μM, 10 μM, 50 μM and 100 μM) for 2 days concentration-dependently increased medium glucose concentrations (Figure 1B, 1C) ; on day 6 and day 8 of induction, however, only 100 μM of EGCG markedly increased medium glucose concentrations, while other low concentrations of EGCG did not work (Figure 1D, 1E), indicating that preadipocytes are more susceptible to EGCG than mature adipocytes. These data demonstrated that EGCG has more robustly inhibitory effects on glucose uptake in preadipocytes than mature adipocytes.

### EGCG inhibits the lipid accumulations during adipogenesis

**Figure 2:**
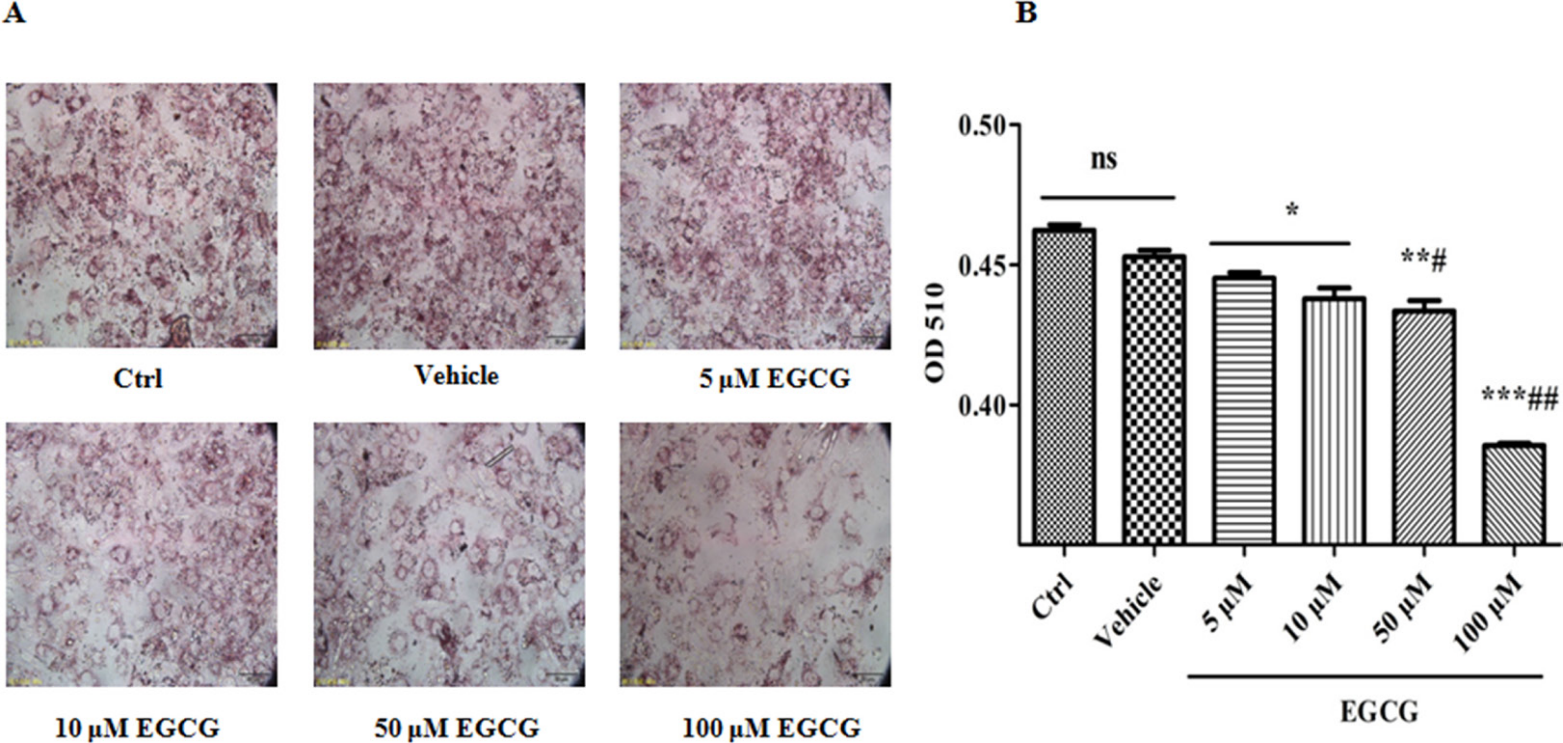
EGCG inhibits the lipid accumulations during adipogenesis 90% confluent 3T3-L1 preadipocytes were induced by DMI for 8 days, during which, the cells were treated with EGCG (5 μM, 10 μM, 50 μM and 100 μM) or DMSO for the first 2 days. Lipid accumulations were visualized and quantified by oil red O staining (**A**, **B**). Error bars show the S.E.M. of 3 independent experiments. ^*^P < 0.05, ^**^P < 0.01, ^***^P < 0.001, vs. Ctrl or Vehicle group; ^#^*P* < 0.05, ^##^*P* < 0.01, vs. EGCG (5 μM). Ctrl: Control, Vehicle: DMSO, ns: no significance, scale bar: 50 μm.

One of important hallmarks of mature adipocytes is the capability of accumulating lipids. Therefore, we sought to detect the capability by staining with oil red O dye. As shown in Figure 2, after 8 days of induction, 3T3-L1 cells showed numerous lipid droplets. With increases of EGCG concentrations, the numbers of lipid droplets progressively decreased, and the minimum occurred in the 100 µM of EGCG. These data suggest that EGCG inhibits lipid accumulation in a concentration-dependent manner.

### EGCG inhibits the cell secretory activity during adipogenesis

**Figure 3:**
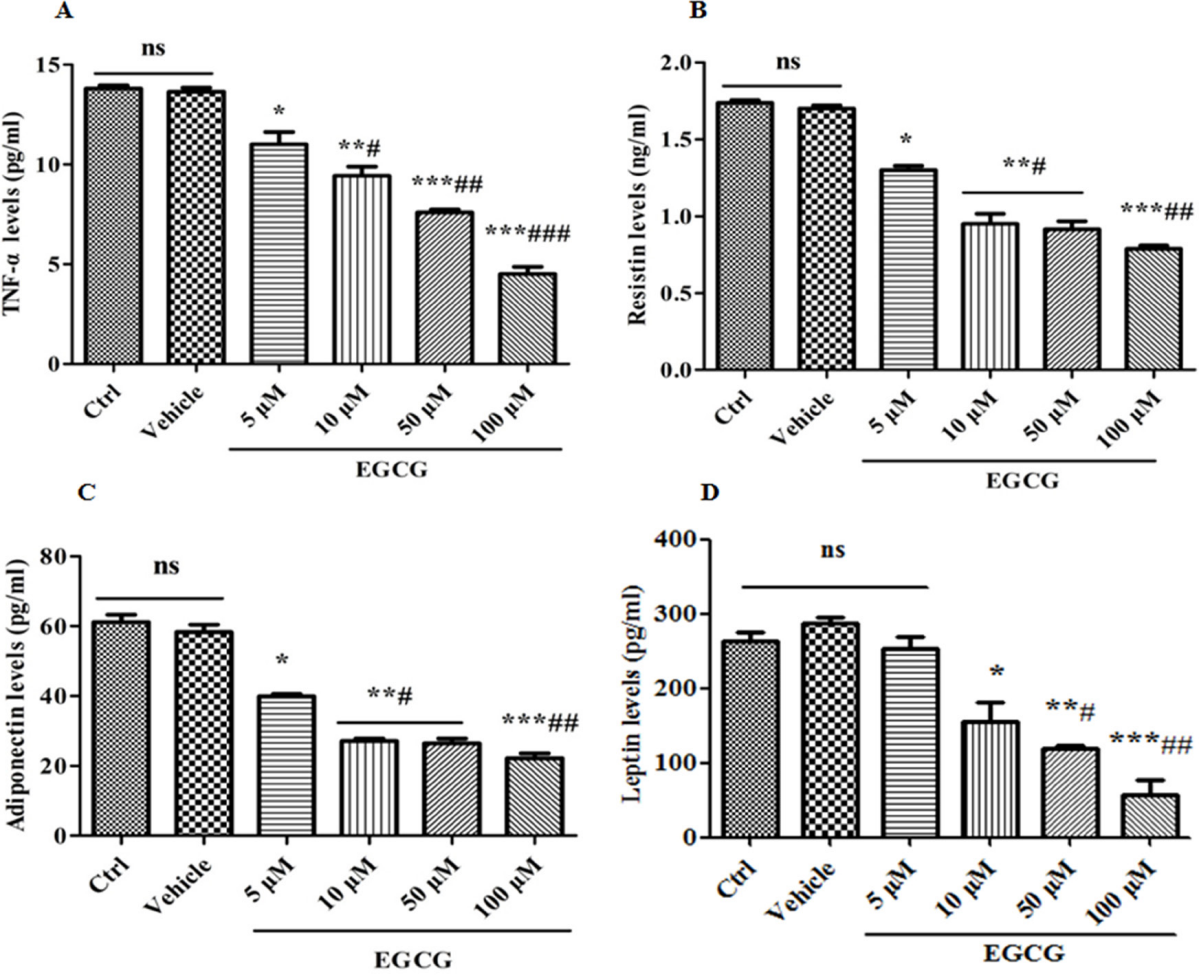
EGCG inhibits the cell secretory activity during adipogenesis 90% confluent 3T3-L1 preadipocytes were induced by DMI for 8 days, during which, the cells were treated with EGCG (5 μM, 10 μM, 50 μM and 100 μM) or DMSO for the first 2 days. The levels of TNF-α (**A**), resistin (**B**), adiponectin (**C**) and leptin (**D**) were determined by ELISA. Error bars show the S.E.M. of 3 independent experiments. ^*^P < 0.05, ^**^P < 0.01, ^***^P < 0.001, vs. Ctrl or Vehicle group; ^#^*P* < 0.05, ^##^*P* < 0.01, ^###^*P* < 0.001, vs. EGCG (5 μM). Ctrl: Control, Vehicle: DMSO, ns: no significance.

Another important hallmark of mature adipocytes is the capability to secrete various adipokines such as TNF-α, adiponectin, resistin, and leptin, etc.. Therefore, we next measured the levels of TNF-α, adiponectin, resistin, and leptin by ELISA. As shown in Figure 3, after 8 days of induction, 3T3-L1 cells secreted considerably high levels of TNF-α, adiponectin, resistin and leptin, implying that the 3T3-L1 cells have differentiated into mature adipocytes. However, EGCG dramatically decreased the secretion levels of TNF-α, adiponectin, resistin and leptin in a concentration-dependent manner, demonstrating that EGCG inhibits the secretory activity of adipocytes during adipogenesis.

### Effects of EGCG on expression levels of PPARγ, FAS, FOXO1 and P-FOXO1 during adipogenesis

**Figure 4:**
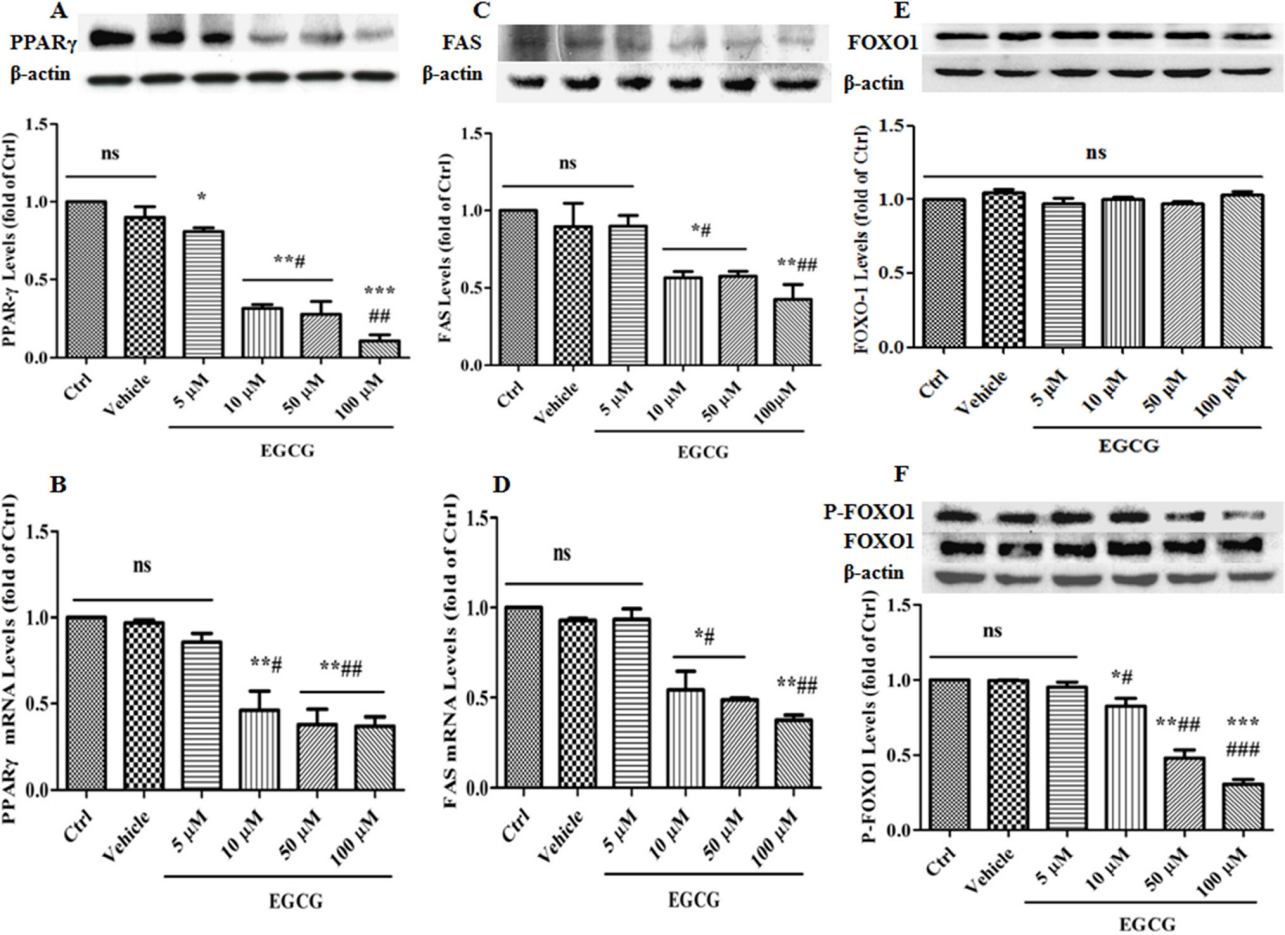
Effects of EGCG on the expression levels of PPARγ, FAS, FOXO1 and P-FOXO1 during adipogenesis 90% confluent 3T3-L1 preadipocytes were induced by DMI for 8 days, during which, the cells were treated with EGCG (5 μM, 10 μM, 50 μM and 100 μM) or DMSO for the first 2 days. The protein levels of PPARγ (**A**), FAS (**C**), FOXO1 (**E**) and P-FOXO1 (**F**) were detected by western blotting, while the mRNA levels of PPARγ (**B**) and FAS (**D**) were determined by quantitative RT-PCR. Error bars show the S.E.M. of 3 independent experiments. ^*^P < 0.05, ^**^P < 0.01, ^***^P < 0.001, vs. Ctrl or Vehicle group; ^#^*P* < 0.05, ^##^*P* < 0.01, ^###^*P* < 0.001, vs. EGCG (5 μM). Ctrl: Control, Vehicle: DMSO, ns: no significance.

It is well known that PPARγ plays a positive role in adipocyte differentiation while FOXO1 does a negative role by inhibiting PPARγ and FAS. Once phosphorylated by its upstream kinases, such as AKT, FOXO1 translocates into cytoplasm from nucleus and its inhibitory effects on PPARγ and FAS are relieved. Hence, we further observed the effects of EGCG on the expression levels of PPARγ, FAS, FOXO1 and P-FOXO1. As anticipated, EGCG down-regulated the expression of PPARγ and FAS at both mRNA and protein levels in a concentration-dependent manner (Figure 4A–4D); for the expression levels of FOXO1 and P-FOXO1, EGCG significantly down-regulated the expression levels of P-FOXO1 in a concentration-dependent manner while it had no notable effect on those of FOXO1 (Figure 4E, 4F). Taken together, these data suggest that EGCG inhibits the differentiation maturation of 3T3-L1 cells induced by DMI through a FOXO1-mediated negative regulation manner.

### Effects of PI3K re-activation on glucose uptake inhibited by EGCG during adipogenesis

**Figure 5:**
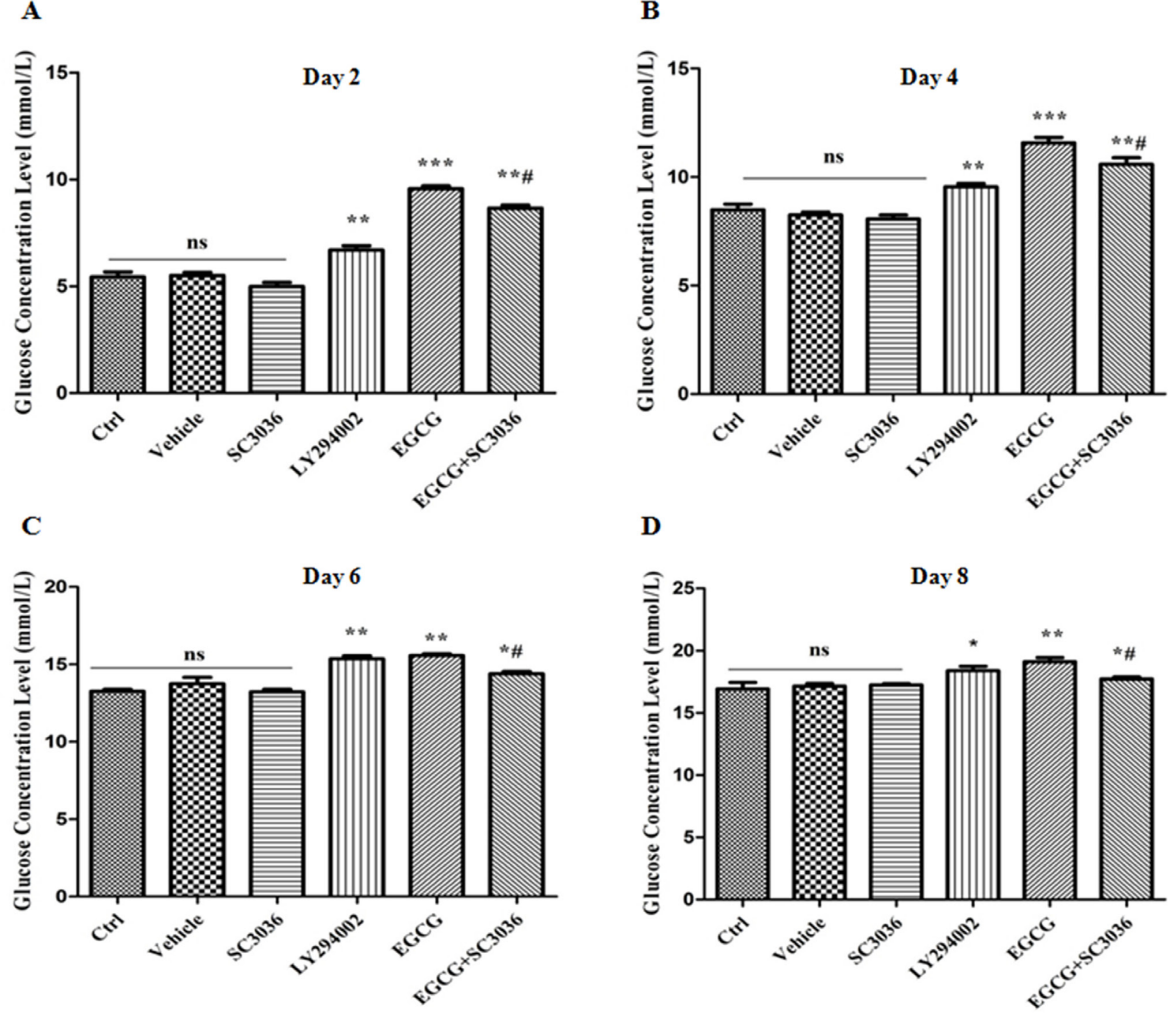
Effect of PI3K re-activation on glucose uptake inhibited by EGCG during adipogenesis 90% confluent 3T3-L1 preadipocytes were induced by DMI for 2, 4, 6 and 8 days, respectively. During induction, the cells were treated either with SC3036 (10 µM, PI3K activator), LY294002 (10 µM, PI3K inhibitor), EGCG (100 µM) alone or co-treated with them for the first 2 days (**A–D**). Besides, the DMSO-treated 3T3-L1 cells were considered as Vehicle. Medium glucose concentrations (mM) were detected with a glucose concentration assay kit. Error bars show the S.E.M. of 3 independent experiments. ^*^P < 0.05, ^**^P < 0.01, vs. Ctrl or Vehicle group; ^#^*P* < 0.05, vs. EGCG (100 μM). Ctrl: Control, Vehicle: DMSO, ns: no significance.

Our previous results have confirmed that EGCG inhibits adipogenesis via negatively regulating the PI3K-AKT signaling. Accordingly, we next wondered whether the inhibitory effect of EGCG on glucose uptake is also via the PI3K-AKT signaling. To address this issue, 10 μM of SC3036 (PI3K activator) and 10 μM of LY294002 (PI3K inhibitor) were utilized. Our present results showed that treatment with 100 μM of EGCG noticeably inhibited the glucose uptake or consumption, presenting the medium glucose concentration markedly elevated from day 2 to day 8 of induction. Interestingly, treatment with LY294002 (10 μM) obtained the same effect as EGCG. However, co-treatment of SC3036 (10 μM) with EGCG (100 μM) reversed the changes caused by EGCG (Figure 5A–5D). Together, these results reveal that the inhibitory effect of EGCG on glucose uptake is abolished by re-activation of PI3K during adipogenesis.

### Effect of PI3K re-activation on lipid accumulation and secretory activity inhibited by EGCG during adipogenesis

**Figure 6:**
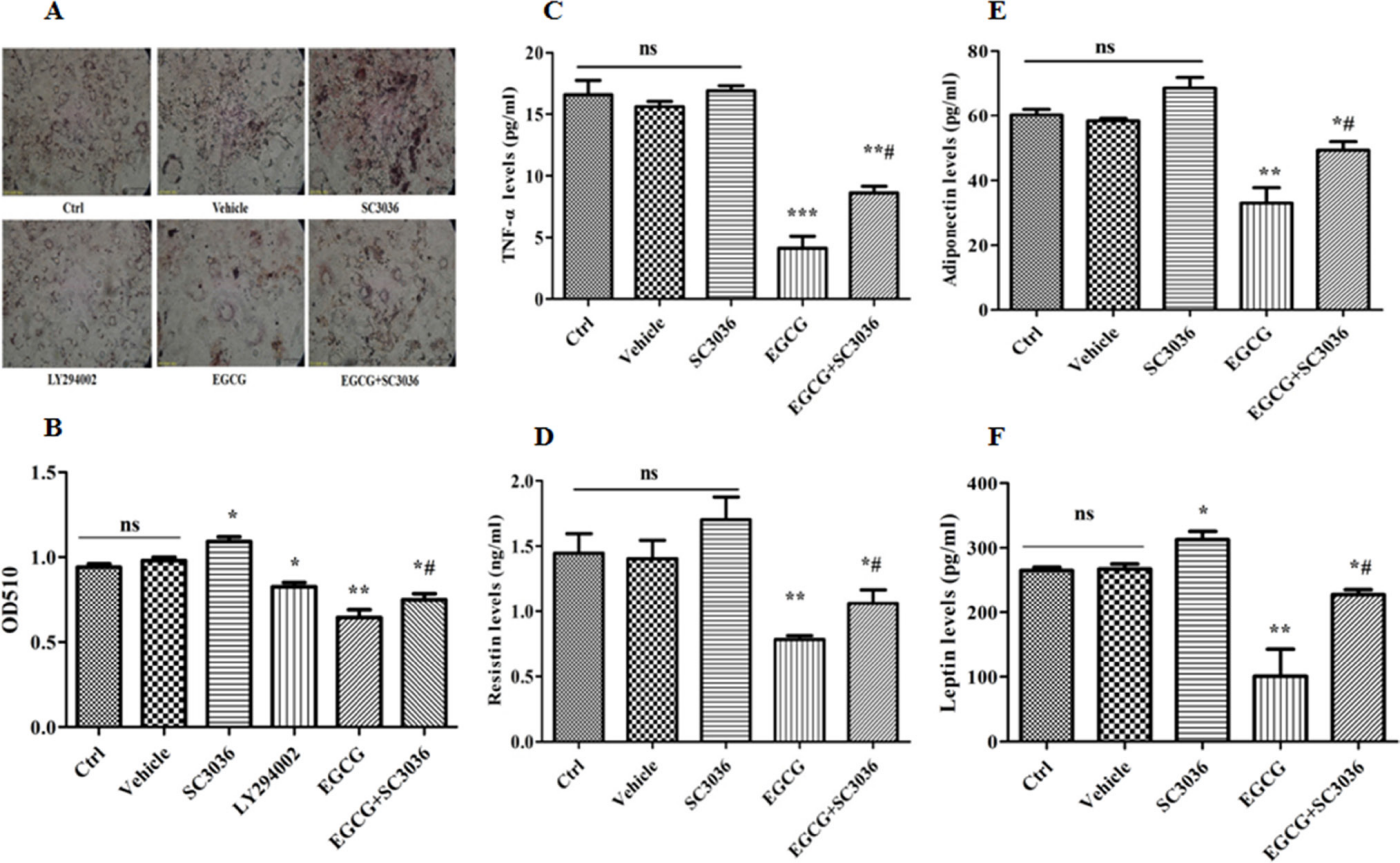
Effect of PI3K re-activation on lipid accumulation and secretory activity inhibited by EGCG during adipogenesis 90% confluent 3T3-L1 preadipocytes were induced by DMI for 8 days. During induction, the cells were treated either with SC3036 (10 µM, PI3K activator), LY294002 (10 µM, PI3K inhibitor), EGCG (100 µM) alone or co-treated with them for the first 2 days Besides, the DMSO-treated 3T3-L1 cells were considered as Vehicle. Lipid accumulations were visualized and quantified by oil red O staining (**A**, **B**). The levels of TNF-α (**C**), resistin (**D**), adiponectin (**E**) and leptin (**F**) were determined by ELISA. Error bars show the S.E.M. of 3 independent experiments. ^*^P < 0.05, ^**^P < 0.01, ^***^P < 0.001, vs. Ctrl or Vehicle group; ^#^*P* < 0.05, vs. EGCG (100 μM). Ctrl: Control, Vehicle: DMSO, ns: no significance, scale bar: 50 μm.

As mentioned above, lipid accumulation and secretory activity are two important functions of mature adipocytes. Therefore, we further sought to evaluate the effects of PI3K re-activation on lipid accumulation and secretory activity inhibited by EGCG. As expected, treatment with 100 μM of EGCG considerably inhibited the lipid accumulation and the secretory activity of adipocytes, presenting the amount of lipids and the levels of TNF-α, adiponectin, resistin and leptin reduced significantly. Interestingly, treatment with LY294002 (10 μM) obtained the same effect as EGCG. Conversely, exposure of 3T3-L1 cells to SC3036 (10 μM) and EGCG (100 μM) restored the alterations caused by EGCG alone (Figure 6A–6F), suggesting that the inhibitory effect of EGCG on lipid accumulation and secretory activity is abrogated by re-activation of PI3K during adipogenesis.

### Effect of PI3K re-activation on the expression levels of PPARγ, FAS, FOXO1 and P-FOXO1 by EGCG during adipogenesis

**Figure 7:**
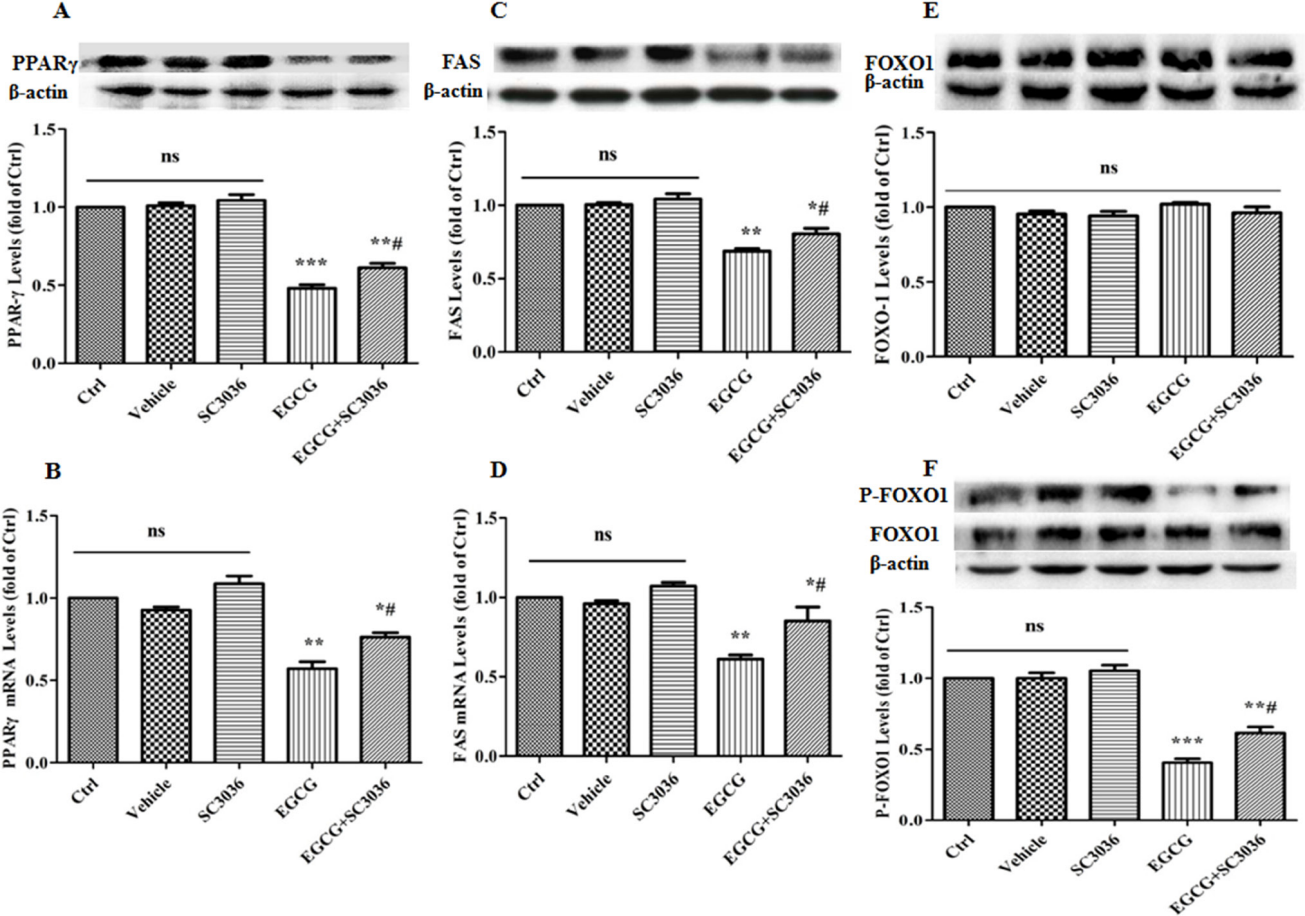
Effect of PI3K re-activation on the expression levels of PPARγ, FAS, FOXO1 and P-FOXO1 by EGCG during adipogenesis 90% confluent 3T3-L1 preadipocytes were induced by DMI for 8 days. During induction, the cells were treated either with SC3036 (10 µM, PI3K activator), EGCG (100 µM) alone or co-treated with them for the first 2 days. Besides, the DMSO-treated 3T3-L1 cells were considered as Vehicle. The protein levels of PPARγ (**A**), FAS (**C**), FOXO1 (**E**) and P-FOXO1 (**F**) were detected by western blotting, while the mRNA levels of PPARγ (**B**) and FAS (**D**) were determined by quantitative RT-PCR. Error bars show the S.E.M. of 3 independent experiments. ^*^P < 0.05, ^**^P < 0.01, ^***^P < 0.001, vs. Ctrl or Vehicle group; ^#^*P* < 0.05, vs. EGCG (100 μM). Ctrl: Control, Vehicle: DMSO, ns: no significance.

Finally, we tested the effect of PI3K re-activation on the expression levels of PPARγ, FAS, FOXO1 and P-FOXO1 by EGCG. As anticipated, treatment with EGCG (100 μM) notably down-regulated the expression levels of PPARγ, FAS and P-FOXO1 rather than FOXO1. In contrast, co-treatment of SC3036 (10 μM) with EGCG (100 μM) markedly antagonized the alterations caused by EGCG alone (Figure 7A–7F). Overall, these data indicate that EGCG inhibits the differentiation and maturation of adipocytes via negatively regulating the phosphorylation of FOXO1 mediated by the PI3K-AKT pathway.

## DISCUSSION

Obesity normally means the increases in the number and size of adipocytes and the imbalance between the intake and expenditure of energy. Adipokines secreted from adipocytes can cause several kinds of obesity-related complications. This research was designed to inhibit adipose differentiation and to lessen the damage caused by redundant adipokines. In order to test the effects of EGCG on glucose uptake and lipid accumulation during adipogenesis, glucose concentration test and oil red O staining were performed. In addition, ELISA was used to detect adipocyte’s secretion function. Our present results clearly demonstrate that EGCG inhibits the differentiation and functions of 3T3-L1 preadipocytes through negatively regulating the phosphorylation of FOXO1 mediated by the PI3K-AKT pathway.

Numerous studies have shown that obesity results from excessive energy intake [33–35]. Our previous study have demonstrated that high glucose and high fat diet induces obesity and insulin resistance in rats [36]. The excess energy is generally stored in adipose tissue in the form of triglyceride (TG), and the higher the amount of TG, the more hypertrophic cells are [37]. Consequently, the amount of TG was observed and quantified by oil red O staining. The present results have shown that EGCG hinders the glucose uptake and the aggregation of lipid droplets during adipogenesis.

There is growing evidence about obesity and its related pathological complications, which makes people aware of the role of adipocytes as a potential participant in controlling physiological and pathological procedure [11, 38]. Adipocytes secrete hundreds of bioactive molecules, known as adipokines, and the levels of adipokines increase during adipocyte differentiation [39]. Some of the adipokines, including leptin, adiponectin, resistin and visfatin can work as hormones to directly regulate the patho-physiological process, and others, such as TNF-α, IL-6 and so on, are involved in immunity and inflammation. In our present study, TNF-α, resistin, adiponectin and leptin were regarded as representative adipokines and their levels were detected to reflect the secretory function of mature adipocytes. Our present results also showed that the secretion levels of TNF-α, adiponectin, resistin and leptin were significantly inhibited by EGCG, suggesting that EGCG inhibits the differentiation of 3T3-L1 preadipocytes into mature adipocytes.

A large number of studies have shown that PPARγ is indispensable in the process of adipocyte differentiation and maturation, and FAS plays a decisive role in the process of fatty acid synthesis and lipid droplet aggregation [40–42]. For example, Kumar R. *et al.* found that fruit extracts of momordica charantia enhance GLUT-4 mediated-glucose uptake by the up-regulation of PI3K and PPARγ [43]. Li Y. *et al.* have also confirmed that 4-Hydroxyderricin, a PPARγ agonist, promotes GLUT-4 and adiponectin expression and also facilitates lipid accumulation in 3T3-L1 cells [44]. Since PPARγ commonly plays as a trigger, while FAS works as a terminal determinant in the process of adipogenesis, PPARγ and FAS were commonly considered as molecule markers of mature adipocytes. In this study, our present data showed that EGCG down-regulated the expression of PPARγ and FAS at both mRNA and protein levels in a concentration-dependent manner, and further confirmed that EGCG inhibits the differentiation of 3T3-L1 preadipocytes into mature adipocytes.

Although many studies have shown that EGCG inhibits the differentiation of 3T3-L1 cells, its underlying mechanism is not fully elucidated. Our previous study has validated that EGCG inhibits adipogenesis through a down-regulation of PPARγ and FAS expression mediated by PI3K-AKT signaling in 3T3-L1 cells [32], but the effect of EGCG on the down-stream events of PI3K-AKT signaling is still elusive. It is reported that FOXO1 is just right a down-stream player of PI3K-AKT signaling. FOXO1 plays a connector between PI3K-AKT and PPARγ [45]. It is generally recognized that FOXO1 binds to the promoter of PPARγ and inhibits PPARγ transcriptional activity in nucleus. Once phosphorylated by AKT, an up-stream kinase of FOXO1, FOXO1 translocates into cytoplasm from nucleus and its inhibitory effects on PPARγ and FAS are relieved [29].

Interestingly, FOXO1 is not only phosphorylated by AKT, but also by MAPK [46] and Wnt etc. (Wnt signaling shares common targets with FOXO1 signaling) [25]. As a consequence, FOXO1 is extensively involved in the negative regulation of cell proliferation, differentiation and cell cycle [47]. Our present results showed that EGCG down-regulated the expression levels of P-FOXO1 in a concentration-dependent manner, while it had no significant impact on those of total FOXO1. These results suggest that EGCG inhibits the differentiation maturation of 3T3-L1 cells induced by DMI through a FOXO1-mediated negative regulation manner.

Since FOXO1 is not only phosphorylated by AKT, but also by MAPK and Wnt. Thus, we ultimately wondered whether the effect of EGCG on FOXO1 is mediated by the PI3K-AKT pathway. To address this question, SC3036, a PI3K activator, was used. Our present data showed treatment with EGCG notably down-regulated the expression levels of PPARγ, FAS and P-FOXO1; conversely, co-treatment of SC3036 with EGCG markedly combated the alterations caused by EGCG or LY294002 alone. Taken together, our findings reveal that EGCG inhibits the differentiation and the maturation of adipocytes via negatively regulating the phosphorylation of FOXO1 mediated by the PI3K-AKT pathway.

Although our present data have identified that EGCG suppresses the differentiation of adipocytes via inhibiting the phosphorylation of FOXO1 mediated by PI3K-AKT signaling, while other possible mechanisms need to be further researched. For instance, as described above, FOXO1 also affect the cell cycle through the regulation of cell differentiation. Consequently, we will further investigate the effects of EGCG on cell cycle-related protein P21 and P27. Besides, the current study is only an *in vitro* research, and an *in vivo* research is also necessary in the future.

In summary, the present study demonstrated that EGCG inhibits the uptake of glucose, aggregation of lipid droplets and secretion of adipokines in a concentration- dependent manner during adipogenesis, suggesting that EGCG inhibits adipocyte’s differentiation, maturation and functions. Furthermore, EGCG also down-regulates the expression levels of PPARγ, FAS and P-FOXO1, while has no significant influence on the expression of FOXO1. Conversely, the activator of PI3K reverses these changes caused by EGCG, which suggests that the inhibitory effects of EGCG might be mediated by the PI3K-AKT-FOXO1 pathway to negatively regulate the expression of PPARγ and FAS. The findings will provide a solid foundation for EGCG and its analogues to prevent and cure obesity-associated diseases.

## MATERIALS AND METHODS

### Material

All chemicals were obtained commercially. High-glucose Dulbecco’s modified Earle’s medium (H-DMEM) and penicillin/streptomycin were purchased from Gibco-BRL (NY, USA). Antibodies against FOXO1, FAS and β-actin were purchased from Abcam (NY, USA). Other antibodies against PPARγ and P-FOXO1 were purchased from Cell Signaling Technologies (MA, USA). All these antibodies were freshly prepared. Glucose concentration assay kit was purchased from Jiancheng (Nanjing, China). Human recombinant insulin, dexamethasone (DEX), and isobutylmethylxanthine (IBMX) were purchased from Sigma Aldrich (MO, USA). Oil red O dye was purchased from Solabio Co. (Beijing, China). EGCG was dissolved in DMSO. EGCG and other chemicals used here were purchased from Solarbio Co. (Beijing, China) unless otherwise specified.

### Cell culture and differentiation

3T3-L1 preadipocytes (American Type Culture Collection, ATCC, USA) were cultured in H-DMEM (4.5 g/L of glucose) supplemented with 10% FBS (fetal bovine serum). DMI, a differentiation cocktail, was used to induce adipocyte differentiation, which combines with insulin (final concentration 10 μg/ml), IBMX (final concentration 0.5 mM) and DEX (final concentration 1 μM) [48]. After the cells reached 90% confluence, they were treated with DMI induction (Induction I) for the first 2 days. Thereafter, the mediums were replaced by Induction II containing insulin only for the next 2 days and by standard culture media for the last 4 days. All cells were cultured and differentiated at 37°C in a humidified environment with 5% CO_2_.

### Experimental protocols

3T3-L1 cells were cultured and differentiated following the procedures as described above. 90% confluent 3T3-L1 preadipocytes were induced by DMI for 2, 4, 6 and 8 days, respectively. During induction, the cells were treated with EGCG (5 μM, 10 μM, 50 μM and 100 μM) or DMSO for the first 2 days. In addition, another batch of 90% confluent 3T3-L1 preadipocytes were induced by DMI for 8 days. During induction, the cells were treated either with SC3036 (10 µM, PI3K activator), LY294002 (10 µM, PI3K inhibitor), EGCG (100 µM) alone or co-treated with them for the first 2 days. Besides, the DMSO-treated 3T3-L1 cells were considered as Vehicle. Finally, medium glucose concentration, lipid accumulation, the levels of TNF-α, resistin, adiponectin and leptin, the expression of FOXO1, P-FOXO1, PPARγ and FAS were detected, respectively.

### Glucose concentration test

Medium glucose concentrations were detected by glucose concentration assay kit from Jiancheng (NanJing, China). Before substituted, the medium was collected on day 2, day 4, day 6 and day 8. After centrifugation (2500 r/min) for 10 min, the supernatants were collected and regarded as samples. The samples (3 μl) were added to working solution (300 μl). ddH2O (3 μl) was added to the working solution (300 μl) and considered as the blank. Glucose (5.55 mM, 3 μl) was added the working solution (300 μl) as a calibration. The working solution is composed of reagent I (phenol, 10.6 mM PH 7.0) and reagent II (PBS, 70 mM; 4-Aminoantipyrine 0.8 mM; glucose oxidase >10 U/ml, peroxidase >1 U/ml PH 7.0). Quantification was performed by microplate reader (Bio-Rad Laboratories) at optical density 505 nm.

### Oil red O staining and quantification

After 8 days of differentiation, 3T3-L1 cells in 6-wells plate were stained by oil red O dye. The cells were washed twice with phosphate-buffered saline (PBS, PH = 7.4), and fixed by 10% formaldehyde for 10 min. Then the formaldehyde was removed and the cells were washed twice again with PBS. Fresh filtrated oil red O dye was added and incubated for 1 h at room temperature. Next, the staining solution was removed and the cells were washed with PBS 3 times. Finally, the cells stained by oil-red O were microscopically examined. Besides this gross evaluation, the dyes in the cells were eluted with 100% isopropanol to be quantified. Optical density values at 510 nm (OD510) of the eluted dye were detected by a spectrophotometer (Bio-Rad Laboratories).

### Enzyme linked immunosorbent assay (ELISA)

The levels of TNF-α were measured by using a TNF-α ELISA assay kit following the manufacturer’s instructions (BOSTER, China). Briefly, 100 μl cell supernatants were added to a 96-well polystyrene microplate pre-coated with TNF-α monoclonal antibody and incubated for 1.5 h at 37°C. The liquid was then removed from the plate and 100 μl of biotin-labeled mouse TNF-α antibody were added and incubated for 1 h at 37°C. After gently washing 3 times with washing buffer, 100 μl of ABC working solution were added and incubated for 30 min at 37°C. After gently washing 5 times, 90 μl of TMB substrate solution were added and incubated for 15–20 min at 37°C. Subsequently, 100 μl of TMB stop solution were added and the O.D. values were measured at 450 nm using a microplate reader (Bio-Rad Laboratories, CA, USA). Resistin, adiponectin and leptin levels were measured with the corresponding ELISA kits (Cloud-Clone Corp, USA), and the operation steps were the same as above-mentioned.

### Western blotting analysis

Western blotting analysis was performed according to the method described in our previous report [32] with a minor modification. Briefly, cells were washed twice with PBS, disrupted on ice for 30 min in NP-40 (50 nM Tris (pH 7.4), 1% NP-40, 150 mM NaCl and 40 mM NaF) or RIPA lysis buffer (Thermo Scientific) supplemented with protease and phosphatase inhibitors (Pierce Chemical) and cleared by centrifugation. Protein concentration was determined with a BCA protein quantification kit (Applygen, Beijing, China). Equal amount of protein (35 μg) in cell lysates was separated by SDS-PAGE, transferred to polyvinylidene difluoride (PVDF) membranes, immunoblotted with specific primary antibodies at a 1:1000 dilution, second antibodies at a 1:2000 dilution and detected by chemiluminescence with the ECL detection reagents (Amersham Biosciences).

### Quantitative RT-PCR

Total RNA was extracted with TransZol Up (TransGen Biotech) and then reversely transcribed into cDNA by the FastQuant RT Kit (Tiangen Biotech). The cDNA was subjected to Q-PCR with QuantiNova™ SYBR Green PCR Kit (Qiagen) following the manufacturer’s instructions. Quantitative PCR analysis was performed with 10 μl SYBR Premix, 2 μl cDNA products, 1.4 μl forward primer, 1.4 μl reverse primer and 5.2 μl nuclease-free water. PCR (90°C for 58 s, 60°C for 34 s, for 40 cycles) was performed with specific primers (PPARγ, 5′-ATTGAGTGCCGAGTCTGTGG-3′ and 5′-GCAAGGCACTTCTGAAACCG-3′;FAS,5′-T TGCTGGCTCACAGTTAAGAG-3′ and 5′-TTCAGGTT GGCATGGTTGAC-3′; GAPDH, 5′-TGGCCTTCCGTGT TCC TAC-3′ and 5′-GAGTTGCTGTTGAAGTCGCA-3′). After PCR amplification, the Ct value was quantified. Target gene mRNA levels were normalized to GAPDH by using the 2 −ΔΔCt method.

### Statistical analysis

Statistical analyses were performed with GraphPad Prism version 5.0 (GraphPad, San Diego, CA, USA). All data were expressed as the means ± S.E.M. Significance was tested with one-way ANOVA and homogeneity test of variance. *P* values < 0.05 were considered statistically significant.

## Abbreviations

AKT: protein kinase B
EGCG: epigallocatechin-3-gallate
ELISA: enzyme linked immunosorbent assay
ERK: extracellular signal-related kinase
FAS: fatty acid synthase
FOXO1: forkhead box-O1
GLUT-4: glucose transporter type 4
IL-6: interleukin-6
MAPK: mitogen-activated protein kinase
PI3K: phosphatidylinositol 3 kinase
PPARγ: peroxisome proliferator activated receptor γ
P-FOXO1: phosphorylated-FOXO1
TG: triglyceride
TNF-α: tumor necrosis factor-α
